# Supplementary material for: MetaRibo-Seq measures translation in microbiomes
Source: Nat Commun. 2020 Jun 29;11:3268. doi: 10.1038/s41467-020-17081-z (PMC7324362; doi:10.1038/s41467-020-17081-z)
Supplement: Supplementary file 10 — Supplementary Data 7 [file 41467_2020_17081_MOESM10_ESM.zip › File2/Confidence_VeryHigh_Taxonomy/31200_out.krona.html]

Javascript must be enabled to view this page.

members
magnitude
magnitudeUnassigned
count
unassigned
taxon
rank

31200\_out

17

2
17
superkingdom

phylum
17
976

class
17
200643

order
171549
17

family
17
171552

17
838
genus

17
59823

SRS012849\_contig\_number\_33491SRS012849\_contig\_number\_33491SRS018888\_contig\_number\_9209SRS024132\_contig\_number\_37859SRS044535\_contig\_number\_23177SRS046502\_contig\_number\_12555SRS053398\_contig\_number\_5441SRS077194\_contig\_number\_10451SRS078419\_contig\_number\_13694SRS097958\_contig\_number\_19853SRS103987\_contig\_number\_15194SRS104036\_contig\_number\_7850SRS144297\_contig\_number\_7545SRS144506\_contig\_number\_43282SRS144603\_contig\_number\_14369SRS144714\_contig\_number\_22958SRS146764\_contig\_number\_contig-100\_215.206580
species
